# The Treatment of Hydrocele

**Published:** 1890-07

**Authors:** 


### THE TREATMENT OF HYDROCELE.

---

Recently there has been a good deal in the journals about the treatment of hydrocele, but, as usual, there is a lack of unanimity of opinion as to the best method. With hydrocele, as with all affections that are amenable to a number of different procedures, there will always be a diversity of opinion as to which is the best, and each operator will select that method with which he is most familiar, or which has proven most successful in his hands.

In simple cases of hydrocele, however, there seems to be a decided sentiment in favor of the injection of pure carbolic acid as proposed by Dr. Levis, of Philadelphia. The arguments urged in favor of this plan of treatment are that it is less painful, easier practiced and more efficacious than any of the other injection methods. Dr. Keyes, of New York, and many of the other leading genito-urinary surgeons accord this method of treatment the highest praise. The method of performing the operation is simple and the necessary instruments always at hand. All that is necessary in the way of instruments is an ordinary hypodermic syringe with two needles, or better one needle, and a small trocar and canula. The hypodermic needle should always be introduced first and a part of the hydrocele fluid drawn off to be sure that it is in the sack, then the remainder of the fluid should be drawn off with a small trocar and canula. After all the fluid has been withdrawn the pure carbolic acid should be injected through the hypodermic needle and thoroughly rubbed around so as to bring it in contact with every part of the sack, or the operation will prove a failure. The scrotum around the needle should be greased with vaseline to prevent the carbolic acid from excoriating the integument. All cases of simple hydrocele may be cured by this simple operation, if done with due regard to detail.

